# To Every Rule There is an Exception: A Rational Extension of Loewenstein's Rule

**DOI:** 10.1002/anie.202013256

**Published:** 2021-01-22

**Authors:** Magnus Fant, Mattias Ångqvist, Anders Hellman, Paul Erhart

**Affiliations:** ^1^ Department of Physics Chalmers University of Technology Gothenburg Sweden; ^2^ Department of Physics and Competence Centre for Catalysis Chalmers University of Technology Gothenburg Sweden

**Keywords:** Al distribution, catalysis, Loewenstein rule, zeolites

## Abstract

Loewenstein's rule, which states that Al−O−Al motifs are energetically unstable, is fundamental to the understanding and design of zeolites. Here, using a combination of electronic structure calculations and lattice models, we show under which circumstances this rule becomes invalid and how it can be rationally extended using the chabasite framework for demonstration.

## Graphical abstract

According to Loewenstein's rule Al−O−Al motifs are energetically unstable in zeolites; here, using atomic scale simulations, the limitations of this rule are demonstrated and a rational extension is shown.

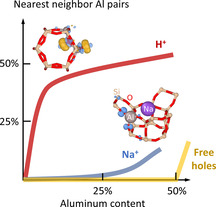

Zeolites are aluminosilicate minerals, used in many different industrial applications, including detergents, adsorbents/desiccants, and catalysts.[^1^, ^2^, ^3^, ^4^] They can occur in a staggering number of frameworks with distinct pore architectures and sizes, which are obtained by different arrangements of the underlying tetrahedral SiO_4_ building blocks.^[5]^ For functionalization, Si^+4^ in the structure are substituted with Al^+3^, where the Al/Si ratio varies from zeolite to zeolite. The substitution introduces a net negative charge into the framework that needs to be balanced by counterions such as H^+^, Na^+^ or Cu^+^. Depending on the character of these counterions, different chemistry is introduced, let it be Brønsted acid chemistry (H^+^) or redox chemistry (Cu^+^/Cu^+2^).

The possibility for zeolites to have different pore sizes makes them ideal for separating various chemicals,[^6^, ^7^] e.g., separation of CO_2_ in natural gas and hydrogen purification, and to enforce shape selectivity in catalytic transformations,^[8]^ e.g., differentiating between linear and branched hydrocarbons. Furthermore, the acidity of the Brønsted sites, whose strength can be tuned by, e.g., isomorphic substitution, plays a crucial role in many hydrocarbon reactions.[^9^, ^10^] Simultaneously, the redox abilities of metal cations in ion‐exchanged zeolites are essential in many oxidation‐reduction reactions, e.g., the selective catalytic reduction of NO_*x*_.^[11,^⋅^12]^

The catalytic performance of the zeolite is, to a large extent, controlled by the distribution of Al^+3^ sites, hence understanding and controlling this distribution is a crucial part of developing more predictive synthesis‐structure‐function relationships.[^13^, ^14^, ^15^, ^16^] The Al distribution is often rationalized using Loewenstein's rule,[^17^, ^18^] which states that Al−O−Al motifs are unstable. This rule is so widely applied and so firmly established that violations warrant special status.[^19^, ^20^, ^21^]

This situation motivates the present study, in which we undertake a critical examination of Loewenstein's rule using the prototypical SSZ‐13 chabasite structure as a model system. While we show that it works as expected when applied in its original context, more importantly, we identify the conditions under which it falls short.

The number of distinct chemical configurations increases exponentially as a zeolite structure is loaded with Al^+3^ and counterions. This combinatorial explosion does not only exhaust any enumeration approach^[22]^ but highlights the importance of configurational entropy. To account for this aspect computationally, we constructed so‐called alloy cluster expansions (CEs),[^23^, ^24^] which provide computationally efficient yet very accurate lattice models for the energy of materials as a function of the chemical distribution. Such models have already been successfully applied to other group 13/14‐group based cage structures.^[25]^

We studied charge compensation by H^+^, Na^+^, K^+^, and Rb^+^ as well as free charge carriers. While the latter compensation mechanism is not available in reality due to the large band gap, it serves as an extreme limit that provides useful insight, as shown below. Initially we considered seven different Wyckoff sites for each counterion. Based on density functional theory (DFT) calculations[^26^, ^27^, ^28^, ^29^] for each site in the dilute limit, we reduced the set of possible sites to four in the case of H^+^ (each associated with one oxygen site) and three in the case of Na^+^, K^+^, and Rb^+^ (located along the channels and in the pore of the chabasite structure; see Supplementary Information for details[^25^, ^30^, ^31^, ^32^, ^33^, ^34^]). Our CE models were then trained to the energies from DFT calculations for approximately 100 to 200 configurations, in which both cell metrics and ionic positions were allowed to relax. The final CEs reproduce these reference data within an average root mean square error over the validation set between 1.7 meV/atom (Rb) and 6.6 meV/atom (free carriers). These models were subsequently sampled by Monte Carlo (MC) simulations in the variance constrained semi‐grand canonical (VCSGC) ensemble^[33]^ to obtain the fraction of Al–Al nearest neighbours (NNs) (i.e. Al−O−Al motifs) as a function of Al content. We intentionally sampled a very wide composition range up to approximately 55 % in order to illustrate the fundamental factors that drive the composition dependence.

The results are strongly dependent on the type of counterion (Figure 1). When charge compensation is achieved by Na^+^, K^+^ or Rb^+^, the fraction of Al–Al NNs is almost zero up to a concentration of 33 %. At high temperatures, a finite, but still small, number of Al–Al NNs is present since they create disorder, which yields an entropic contribution.

**Figure 1:**
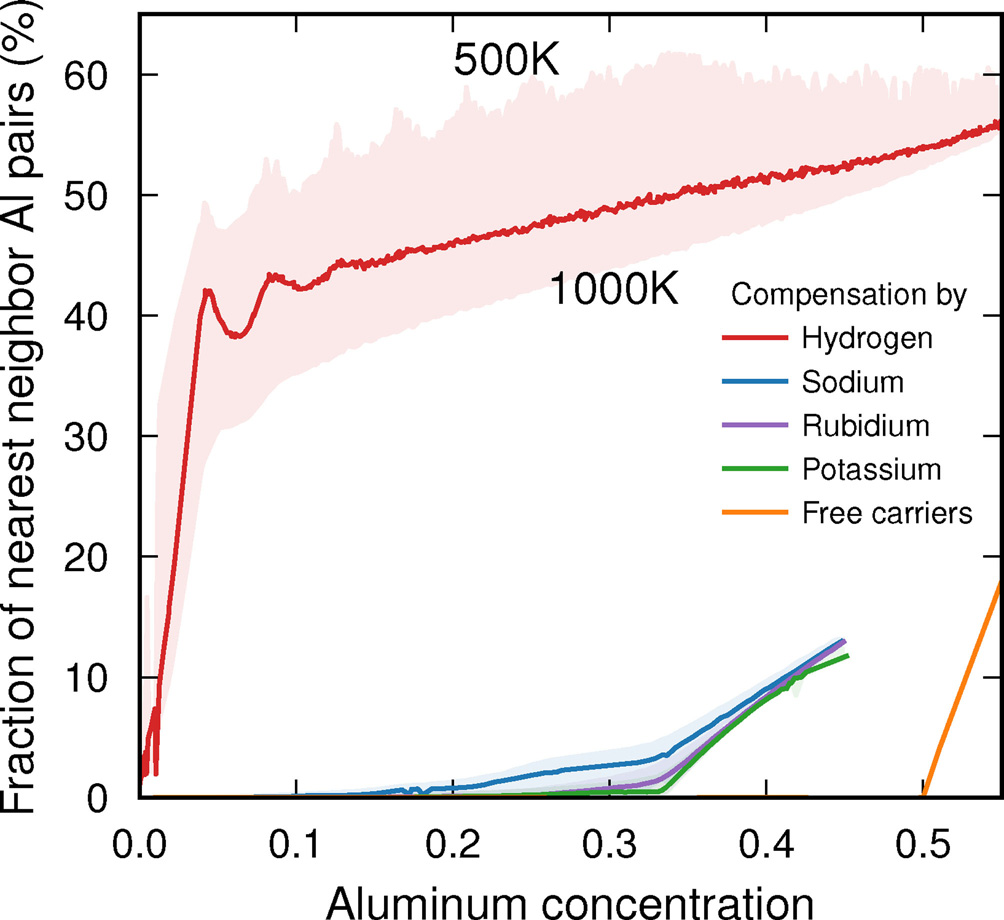
Variation of the fraction of Al–Al nearest‐neighbor pairs as a function of Al content in a chabasite framework (SSZ‐13) using either ions (H^+^, Na^+^, K^+^, Rb^+^) or free carriers for charge compensation.

While the behavior observed for the alkali ions is largely compatible with Loewenstein's rule, hydrogen presents a very different case (Figure 1). The fraction of Al–Al NNs rises sharply at small Al content to about 40 %, after which it continues to increases gradually. This behavior can be traced to the effectively *attractive* interaction between Al^3+^ if charge compensated by H^+^, which was already noted in Ref. [19] and which will be rationalized below in terms of a competition between electrostatics and strain. Notably this attraction leads to the formation of Al “clusters” (Figure 3). The temperature dependence of the number of Al–Al NNs is more pronounced than in the case of alkali counterions, which is consistent with a larger entropic contribution thanks to a larger number of available sites (4 sites with multiplicity 18 vs. 3 sites with multiplicities between 3 and 9) and smaller energy differences between these sites.

Finally, in the case of compensation by free carriers, the fraction of Al–Al NNs is *exactly* zero regardless of temperature up to 50 % Al, at which point it is geometrically impossible to avoid Al–Al NNs.

One thus observes three different types of behavior depending on whether compensation is achieved by H^+^, alkali ions or free carriers. Strictly speaking, Loewenstein's rule is thus only obeyed in the case of free carrier compensation. This begs the question of what factors are at play in the other two cases. To resolve this question, it is instructive to consider the electronic structure of the different counterions in the dilute limit (Figure 2).

**Figure 2:**
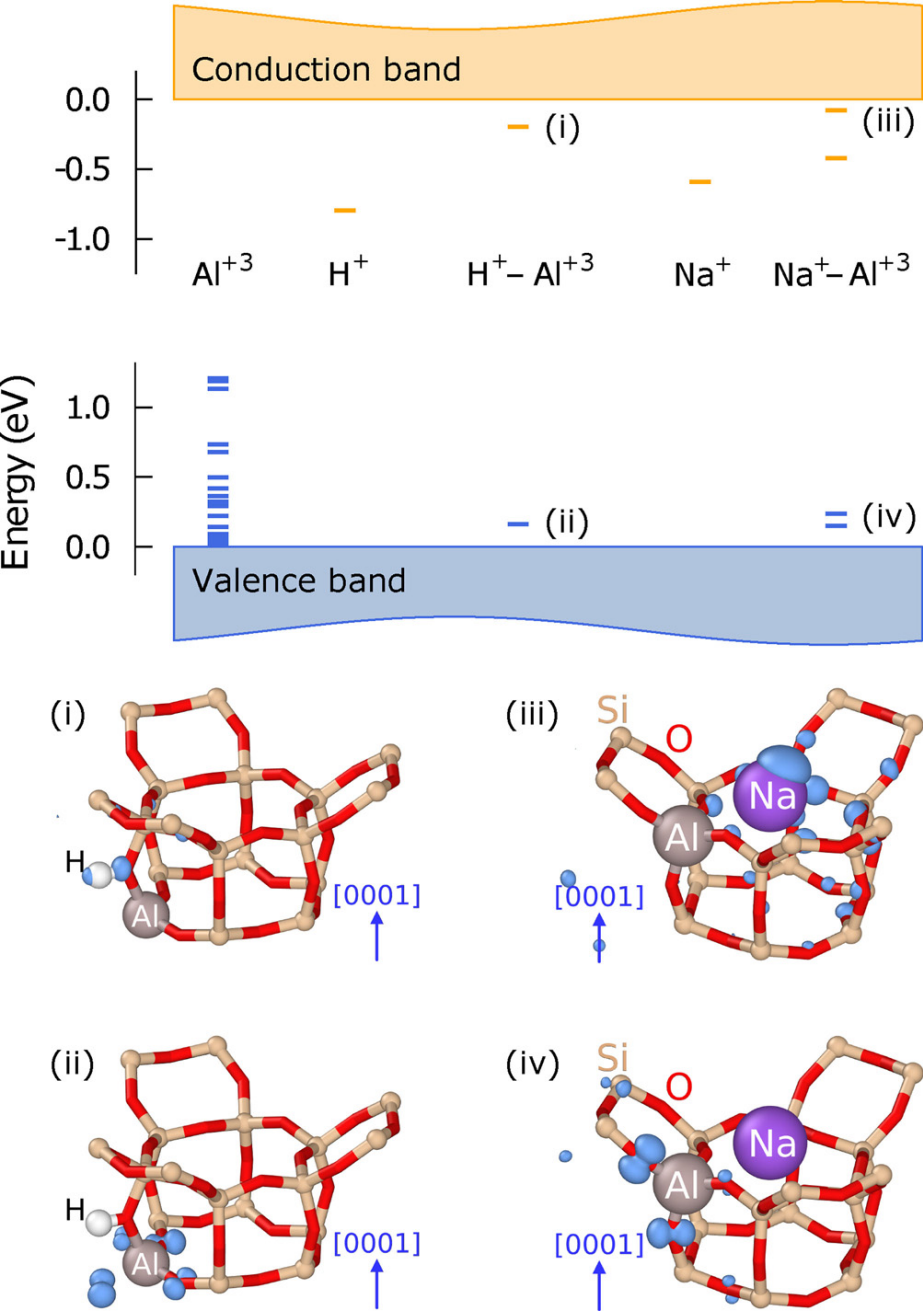
Electronic energy levels associated with different defects in SSZ‐13 (top) and their real space representations shown as isosurfaces (bottom). Occupied and empty levels are shown in blue and orange, respectively. Visualized using ovito.^[35]^

First, we consider the electronic structure of *isolated* impurity ions in SSZ‐13. The exchange of a single Si^+4^ with Al^+3^ (an acceptor in semi‐conductor terminology) leads to the emergence of several levels near the valence band maximum (VBM). These states are semi‐local in character and arise from the hybridization of O‐2p orbitals (similar to the orbital shown in Figure 2 (ii)). In the case of H^+^ and Na^+^ (donors in semi‐conductor terminology), on the other hand, one observes the appearance of deep localized levels near the conduction band minimum (CBM). (K^+^ and Rb^+^ exhibit characteristics similar to Na^+^ and are not discussed here in detail.)

In the lowest energy configuration, H^+^ is situated next to an oxygen site; the lowest unoccupied level is localized on the H^+^ site and exhibits p‐character with an orientation along the H−O bond axis, corresponding to the lowest unoccupied molecular orbital of OH^−^ (similar to Figure 2 (i)). Na^+^, on the other hand, prefers 6c sites, which reside along the axes of the channels of the SSZ‐13 structure. The lowest unoccupied levels exhibit semi‐local character as the corresponding charge density is distributed over several sites surrounding the Na^+^ site (similar to Figure 2 (iii)).

Next, we consider the electronic structure of the compensated systems, in which, generally speaking, the electron from the cation is transferred to unsaturated O−Al bonds, formally turning all framework oxygens into O^−2^. In the case of H^+^–Al^+3^, the attraction between H^+^ and the saturated O^−2^ renders the O‐sites close to Al^+3^ energetically preferred. This configuration leads to two levels in the band gap (see H^+^–Al^+3^ in Figure 2): an empty level near the CBM, which exhibits p‐like character as in the case of the individual H^+^ (Figure 2 (i)), and an occupied level near the VBM, which is comprised of p‐orbitals localized at the four oxygen neighbors of the Al^+3^ site (Figure 2 (ii)).

One observes several localized levels in the band gap also in the case of Na^+^–Al^+3^ (Figure 2). While the levels near the VBM exhibit similar characteristics as in the case of H^+^–O^−2^ (Figure 2 (iii)), the levels in the vicinity of the CBM are much less localized as the charge density is not only located at the Na^+^ site but distributed over sites up to three neighbor shells away (Figure 2 (iii)). This behavior can be attributed to the much larger size of Na^+^, which forces it to occupy sites along the channel, an effect that is even more pronounced for K^+^ and Rb^+^.

Further insight is provided by analyzing the charge density redistribution upon compensation. To this end, we consider the difference between the charge densities of the compensated system, the system with only Al^+3^ ions, and the free counterions in their atomic state. This analysis shows that H^+^–O^−2^ pairing leads to the formation of small dipoles but—crucially—no isolated monopoles (Figure 3 a), which considerably reduces the repulsive interaction between like‐charged species; in other words the leading electrostatic interaction term falls of as 1/*R* ^3^ as opposed to 1/*R*. The insertion of Al gives rise to a notable structural relaxation as the average Al–Si nearest‐neighbor distance is 3.21 Å to be compared with an average Si–Si distance of 3.12 Å in the ideal SSZ‐13 structure. The strain field associated with each Al^+3^ site gives rise to an effective attraction, which in the absence of strong electrostatic repulsion leads to the clustering of Al^+3^ (Figure 3 c,d), a behavior that can also be observed for (effectively) charge‐neutral defects in other materials.^[36]^

**Figure 3:**
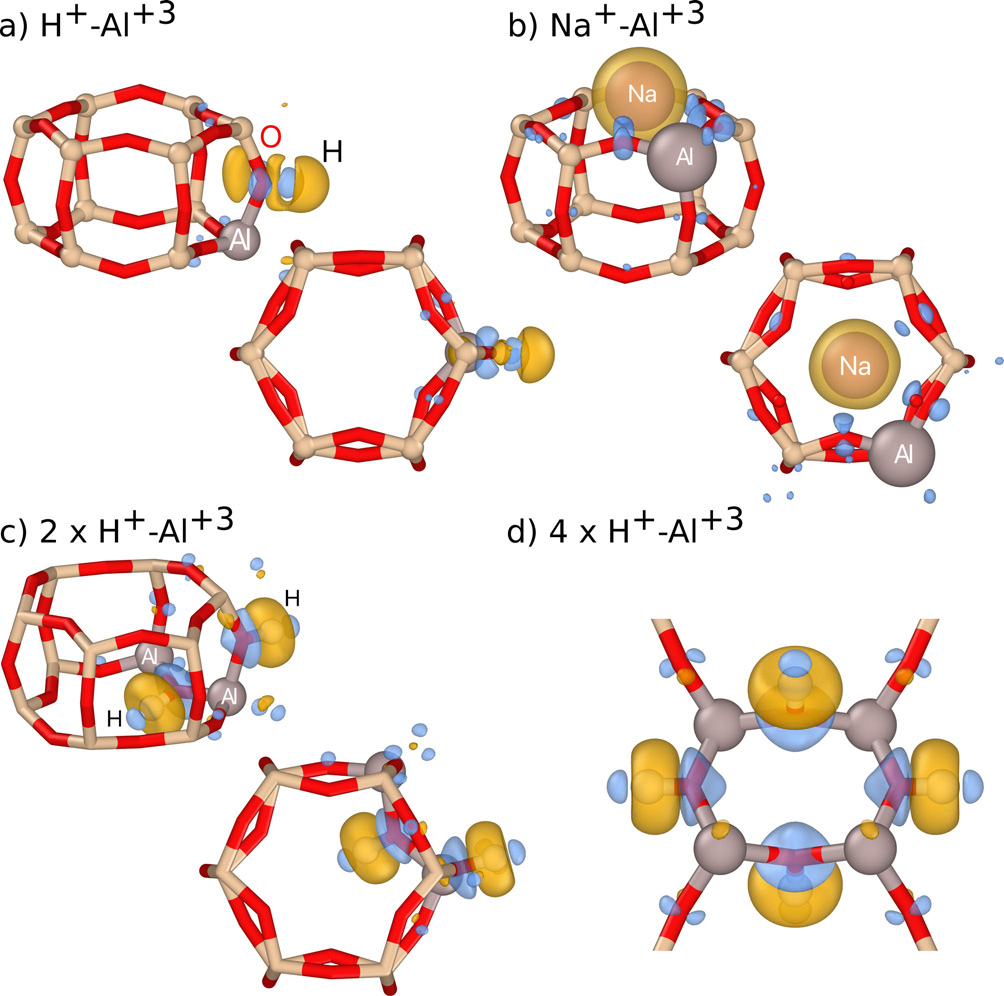
Low energy configurations in perspective (top row) and viewed along the *c*‐axis (bottom row) of (a) H^+^–Al^+3^, (b) Na^+^–Al^+3^, (c) 2×H^+^–Al^+3^, and (d) 4×H^+^–Al^+3^. Yellow and blue isosurfaces show, respectively, negative and positive charge redistribution upon compensation with the different counterions.

In sharp contrast, in the case of Na^+^, one observes a localization of negative charge on the Na^+^ site with the compensating positive charge distributed over the surrounding O^2−^ sites (Figure 3 b). As a result, the strain‐mediated attraction is overruled by electrostatic repulsion (falling of as 1/*R*), preventing Al^+3^ clustering. As the system strives to maximize the separation between equally charged Na^+^ species, the attractive O–Na interaction also forces a separation of Al^+3^ species, since these are indirectly associated with saturated O^−2^. The most extreme form of this behavior is obtained if compensation is achieved via free carriers (Figure 1). In this case, one effectively obtains a system of point charges (distributed over O^−2^ sites) and a homogeneous background charge (corresponding to free carriers), for which the electrostatic energy is minimized by maximizing the Al–Al spacing.

The above analysis suggests that the underlying factors are largely electrostatic in nature and thus relatively insensitive to the framework structure. In fact, calculations of Al clusters in other frameworks constructed in analogy to the ones found for the chabasite framework yield very similar binding energies and show compensation with free carriers and H^+^ to, respectively, prevent and favor Al clustering (see Supporting Information for details).

To summarize, the thermodynamic distribution of Al^+3^ in the prototypical SSZ‐13 zeolite is sensitive to the type of counterion used for charge compensation. While the Al–Al interaction is effectively attractive when compensating with H^+^, it is repulsive in the case of Na^+^, K^+^ or Rb^+^. This difference can be understood by considering counterion size, level of charge localization, and local structural rearrangements. In particular, H^+^ counterions enable full saturation of O^−2^ and very localized charge compensation, which effectively reduces the strong electrostatic repulsion between monopoles. This allows the strain‐mediated attraction between Al^+3^ sites to take over, leading to clustering, and hence violation of the Loewenstein rule. By contrast, the larger size of Na^+^, K^+^ or Rb^+^ prevents the formation of localized bonds with O^−2^ sites and the compensation charge is much more delocalized. As a result, repulsive electrostatic interactions govern the Al^+3^ distribution.

While H‐SSZ‐13 is important for applications, H^+^ species are usually not present during synthesis. The Al distribution that is commonly encountered in these materials will thus not correspond to an equilibrium state but is instead preserved kinetically due to the very large barriers for Al redistribution. The present insight raises the question of whether synthesis routes and annealing procedures can be devised that exploit the mechanisms described above to control the Al distribution more consciously. Here, lattice geometry and the locations of counterion sites play important roles. While the channels and pores in SSZ‐13 are very small, it could be possible to realize local charge compensation more efficiently in the presence of larger channels and pores also with cations larger than H^+^.

Finally, we note that the present approach adds another computational method to the toolbox available for understanding and designing zeolite structure and chemistry.[^14^, ^16^, ^37^] Future work in this direction should address for example the distribution of divalent and trivalent species,[^1^, ^38^, ^39^] molecular counterions as well as other frameworks.

## Conflict of interest

The authors declare no conflict of interest.

## Supporting information

As a service to our authors and readers, this journal provides supporting information supplied by the authors. Such materials are peer reviewed and may be re‐organized for online delivery, but are not copy‐edited or typeset. Technical support issues arising from supporting information (other than missing files) should be addressed to the authors.

Supplementary
