# Supplementary material for: To Every Rule There is an Exception: A Rational Extension of Loewenstein's Rule
Source: Angew Chem Int Ed Engl. 2021 Jan 22;60(10):5132–5. doi: 10.1002/anie.202013256 (PMC7986852; doi:10.1002/anie.202013256)
Supplement: Supplementary file 1 — Supplementary [file ANIE-60-5132-s001.pdf]

Angewandte Chemie
Eine Zeitschrift der Gesellschaft Deutscher Chemiker

Supporting Information

To Every Rule There is an Exception: A Rational Extension of Loewenstein’s Rule

*Magnus Fant, Mattias Ångqvist, Anders Hellman, and Paul Erhart**

anie_202013256_sm_miscellaneous_information.pdf

Contents

Supporting Notes **1**
1. Total energy calculations . . . 1
2. Extension to other frameworks . . . 2
3. Compensation by homogeneous background charge . . . 2
4. Electronic structure calculations . . . 3
5. Alloy cluster expansions . . . 3
6. Monte Carlo simulations . . . 3
7. Data and code availability . . . 4

Supporting References **4**

Supporting Notes

Supporting Note 1: Total energy calculations.

Electronic structure calculations were carried out within density functional theory in the projector augmented wave (PAW) formalism as implemented in the Vienna ab-initio simulation package. Standard PAW setups were employed for Si (4 valence electrons), O (6), H (1), and Na (1); in the case of K (7) and Rb (9) the 3p and 4s/4p semicore states, respectively, were included as well. The PBE functional was employed to describe exchange and correlation [1]. The plane-wave energy cutoff was set to 520 eV throughout. Both atomic positions and cell metric were relaxed until the maximum force fell below 30 meV/angstrom and stresses did not exceed 0.5 GPa. The Brillouin zone was sampled using a $\boldsymbol{k}$-point mesh with a spacing of at most $0.35\,\text{Å}^{-1}$, equivalent to a $2 \times 2 \times 2$ Monkhorst-Pack mesh for the primitive cell.

The initial evaluation of different crystallographic sites for the CHA (chabasite) framework [2], was carried out based on the primitive (36-atom) unit cell (Supporting Figure 1), employing a homogeneous background charge to describe the *isolated* charged species (Al^{+3}, H^+, Na^+, K^+, Rb^+). In the case of H^+, the sites associated with O^{-2} ions (18f, 18g, two types of 18h sites; see Supporting Table 3) to be energetically strongly favored over sites in the channels or pores (3b, 6c, 9e). The latter are at least 1.8 eV higher in energy and are thus energetically prohibitive, whence they have been excluded from the cluster expansion (CE) (see Supporting Note 5). The opposite is observed for the alkaline species (Na^+, K^+, Rb^+), for which the sites that directly neighbor an O^{-2} site (18f, 18g, two types of 18h sites) are even unstable and hence have not been included in the CE.

Reference structures for the construction of alloy CEs for CHA were generated by enumeration [3, 4] for configurations with up to two Al atoms and by randomization over the entire composition range. Calculations for the construction of CEs were primarily carried out using the primitive (36-atom) unit cell. While the Al-rich end of the composition range is not of interest in itself here, we considered the entire concentration range in order to improve the stability and reliability of the CEs derived from these data. In the case of compensation by H^+, Na^+, K^+, and Rb^+ the counterion-to-Al ratio was fixed to one to ensure charge neutrality.

To model compensation by free carriers configurations with only Al (no counterions) were created and charge compensation was achieved by a homogeneous background charge. Since the calculations become unreliable for large background charges, the Al/Si ratio in these cases was limited to be below 3/9 (also see Supporting Note 3).

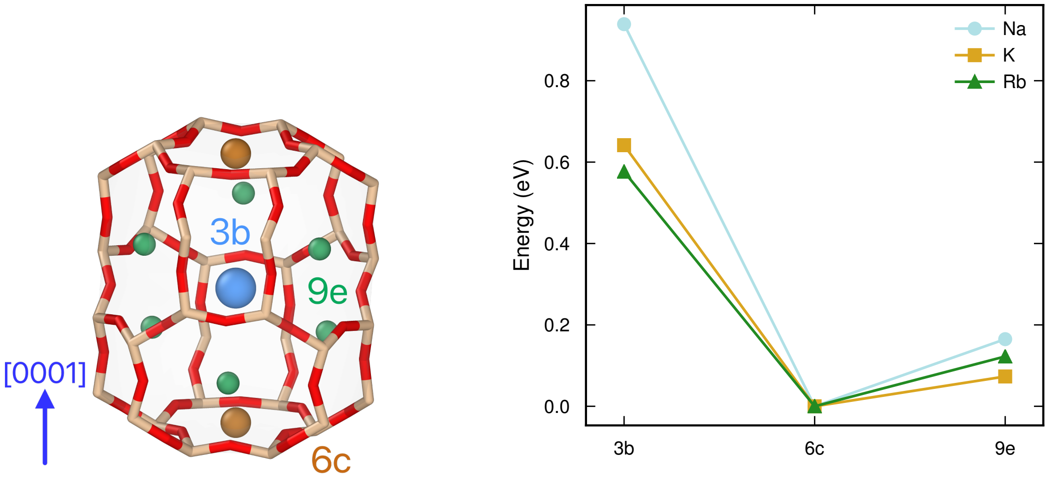

Supporting Figure 1: Left: Location of 3b, 6c, and 9e Wyckoff sites in the SSZ-13 structure. Right: Energy differences between different Wyckoff sites for Na^+, K^+, and Rb^+.

Supporting Note 2: Extension to other frameworks.

To demonstrate that clustering of Al in the presence of H counterions is not specific for the CHA structure, additional calculations were carried out for ACO, MFI, MOR and SOD frameworks, which were retrieved from the Database of Zeolite Structures [2]. For these calculations we increased the primitive unit cell to reach 192 (ACO), 108 (CHA), 288 (MFI), 144 (MOR) and 288 (MOR) sites. We then inserted 2 or 4 Al atoms in the structure, either placed far apart from each other or in the form of a cluster, following the motifs observed for CHA (see Fig. 3 of the main paper). These structures were subsequently relaxed using either a homogeneous background charge or by hydrogen ions, placed at the oxygen sites neighboring the Al sites. Relaxation and evaluation of the total energies of these structures were carried out using the same computational parameters as for the calculations described above. We then computed the binding energies as the total energy difference between clustered and dissociated configurations with negative binding energies indicating that association is favorable. The results demonstrate that the behavior observed in CHA is qualitatively and possibly even partly quantitatively transferable to other zeolite frameworks (Supporting Table 1).

Supporting Table 1: Binding energies in eV of Al clusters obtained via PBE calculations for different framework types.

Compensation by	Free carriers		H^+	
Number of Al	2	4	2	4
ACO	0.58	1.44	−0.16	−0.36
CHA	0.51	1.08	−0.19	−0.36
MFI	0.49	1.31	−0.19	−0.49
MOR	0.46	1.10	−0.13	−0.23
SOD	0.56	1.43	0.01	−0.08

Supporting Note 3: Compensation by homogeneous background charge.

Compensation with a homogeneous background charge is widely employed for treating different charge states of defects in solids. In these cases one is usually interested in the so-called dilute limit, i.e. very small defect concentrations, and the combination of a homogeneous background charge with a localized defect charge state can lead to image charge interactions (along with other finite-size effects) [5]. Since the latter can make a sizable contribution to the total energy, a host of correction schemes has been devised to remove this contribution. The latter is especially large in materials with small dielectric constants and thus little screening (such as zeolites) as well as for large defect charge states (since the correction scales to leading order with the square of the excess charge).

In the present case, we are, however, decidedly not interested in the dilute but the concentrated limit and hence these image interactions are not an artifact but rather an important part of the energetics. We therefore did not apply any corrections and used the as-calculated energies for the construction of the CE. We still limited the Al/Si ratio to be below 3/9 in order to capture potential chemical interactions for smaller Al concentrations. If the full concentration range was sampled (which could also lead to convergence problems in the density functional theory (DFT) self-consistency cycle), one should merely recover the fully ionic picture dominated by electrostatics, leading to the Loewenstein rule in its original form.

Finally, we note that in the case of the explicit counterions, charge compensation is achieved through two (relatively) localized charges and the interaction decays rather quickly. In contrast, in the case of charge-compensation through delocalized charges the interaction is more long-ranged. This is reflected in a longer-ranged CE and a higher cross-validation (CV)-root-mean-square error (RMSE) score.

Supporting Note 4: Electronic structure calculations.

The electronic levels of different species in the CHA framework were calculated using $2 \times 2 \times 2$ (288-atom) supercells to minimize defect-defect interactions and to obtain clean defect signatures. As further validation we carried out calculations using the PBE0 exchange-correlation functional [6]. While the latter, as expected, yields a larger band gap, the positions of the levels relative to the band edges varies by 0.1 eV or less.

Supporting Table 2: Level positions in CHA obtained from PBE and PBE0 calculations using 288-atom supercells.

Configuration	Level		relative to
	PBE	PBE0	
H^+	−0.80	−0.73	CBM
Na^+	−0.59	−0.63	CBM
K^+	−0.51	−0.56	CBM
Rb^+	−0.50	−0.55	CBM
H–Al	0.16	0.18	VBM
	−0.20	−0.21	VBM

Supporting Note 5: Alloy cluster expansions.

Alloy CEs [7] were constructed by automatic relevance determination regression using the ICET package [4] (version 1.3), which uses SCIKIT-LEARN for optimization tasks [8]. Alloy CEs can be written in the general form

$$E = E_0 + \sum_{\alpha} m_\alpha J_\alpha \bar{\Pi}_\alpha(\boldsymbol{\sigma}), \tag{1}$$

where E denotes the energy. The summation runs over all symmetry inequivalent clusters α with multiplicity m_α and effective cluster interaction (ECI) J_α. The cluster correlations α are computed as symmetrized averages of products over the pseudospin vector $\boldsymbol{\sigma}$. The latter represent the occupation of lattice sites by, e.g., Al, H, Na etc. To describe Al as well as counterion distributions, both respective sublattices were included in the construction of the CE. The Wyckoff sites included in each case are shown in Supporting Table 3 and Supporting Figure 1. We note that this approach has already been successfully applied to describe chemical ordering in clathrates, another class of inclusion compounds based on group 13 and 14 elements [9].

A systematic convergence study with respect to clusters to include in the summation was carried out, considering pair terms up to 6.9 Å and triplet terms up to 5.0 Å. The performance of these CE models was evaluated by the cross-validated root mean square error (CV-RMSE), which was computed by k-fold cross-validation (see Ref. 4 for details). The final CEs that were used in the subsequent Monte Carlo (MC) simulations included only pair terms up to a range of 5.5 Å as the inclusion of more terms did not improve the CV-RMSE. CV-RMSE values and other pertinent information regarding the CEs used for MC simulations are compiled in Supporting Table 3.

Supporting Note 6: Monte Carlo simulations.

MC simulations were carried using both the canonical and the variance constrained semi-grand canonical (VCSGC) ensemble [10] as implemented in the MCHAMMER module of ICET. The acceptance probability in the VCSGC is given by (here we adopt the notation in Ref. 11, where further details can be found)

$$\mathcal{P} = \min\left\{1, \exp\left[-\beta\Delta E - \kappa\Delta N_B(\phi + \Delta N_B/N + 2N_B/N)\right]\right\}, \tag{2}$$

where ΔE is the energy change during a trial move, ΔN_B is the change in the number of B species, N is the number of available sites and $\beta = 1/k_B T$ is the inverse temperature. The VCSGC ensemble is controlled via ϕ and κ where κ determines the strength of the constraint on the variance of the concentration while ϕ determines the average concentration. Inspection of the expression above, shows that for $\kappa = 0$ one recovers the canonical ensemble. A full derivation and an example application of the VCSGC ensemble can be found in Refs. 10 and 11, respectively. Since the VCSGC ensemble led to better statistics and higher acceptance probabilities than the canonical ensemble [10], here we only report results from the former.

Simulations were conducted using $2 \times 2 \times 2$ supercells (96 Al/Si sites, 192 O/H sites, 48 Na/K/Rb sites) and run for up to 10^6 MC trial moves. To preserve the counterion-to-Al ratio, a trial move consisted of simultaneously adding (or removing) both an Al and a counterion. Since the binding energy between Al and H is on the order of 1 eV, trial moves were furthermore constrained such that Al–H pairs remained intact. The concentration range was sampled by scanning the ϕ parameter of the VCSGC ensemble from -2.25 to 0.05 while keeping the κ parameter at 100. The number of Al–Al nearest neighbors (NNs) was determined by counting the number of oxygen sites with two Al neighbors. The number of Al–Al NNs was averaged over the MC trajectory to obtain the data shown in Figure 1 of the paper.

Supporting Table 3: Alloy cluster expansions used for MC sampling.

Compensation	Number of reference structures	CV-RMSE (meV/atom)	Singlets	Pairs	Wyckoff sites
H	875	2.7	5	58	18f, 18g, 2× 18h
Na	228	3.0	4	16	3b, 6c, 9e
K	233	1.7	4	14	3b, 6c, 9e
Rb	69	5.4	4	14	3b, 6c, 9e
free carriers	248	6.6	1	7	–

Supporting Note 7: Data and code availability.

The results of the DFT calculations have been compiled in a set of ASE databases [12]. They are available as a Zenodo dataset, which also contains scripts for the construction and sampling of CEs as well as selected results of these simulations [13].

Supporting References

[1] J. P. Perdew, K. Burke, and M. Ernzerhof, *Generalized Gradient Approximation Made Simple,* Physical Review Letters **77**, 3865 (1996). `doi:10.1103/PhysRevLett.77.3865`.

[2] C. Baerlocher and L. McCusker, *Database of Zeolite Structures,* `http://www.iza-structure.org/databases/`.

[3] G. L. W. Hart and R. W. Forcade, *Generating derivative structures from multilattices: Algorithm and application to hcp alloys,* Physical Review B **80**, 014120 (2009). `doi:10.1103/PhysRevB.80.014120`.

[4] M. Ångqvist, W. A. Muñoz, J. M. Rahm, E. Fransson, C. Durniak, P. Rozyczko, T. H. Rod, and P. Erhart, *icet - A Python library for constructing and sampling alloy cluster expansions,* Advanced Simulation and Theory **2**, 1900015 (2019). `doi:10.1002/adts.201900015`.

[5] G. Makov and M. C. Payne, *Periodic boundary conditions in ab initio calculations,* Physical Review B **51**, 4014 (1995). `doi:10.1103/PhysRevB.51.4014`.

[6] C. Adamo and V. Barone, *Toward reliable density functional methods without adjustable parameters: The PBE0 model,* Journal of Chemical Physics **116**, 6158 (1999). `doi:10.1063/1.478522`.

[7] J. M. Sanchez, F. Ducastelle, and D. Gratias, *Generalized cluster description of multicomponent systems,* Physica A: Statistical Mechanics and its Applications **128**, 334 (1984). doi:10.1016/0378-4371(84)90096-7.

[8] F. Pedregosa, G. Varoquaux, A. Gramfort, V. Michel, B. Thirion, O. Grisel, M. Blondel, P. Prettenhofer, R. Weiss, V. Dubourg, J. Vanderplas, A. Passos, D. Cournapeau, M. Brucher, M. Perrot, and E. Duchesnay, *Scikit-Learn: Machine Learning in Python,* Journal of Machine Learning Research **12**, 2825 (2011). http://www.jmlr.org/papers/v12/pedregosa11a.html.

[9] M. Ångqvist and P. Erhart, *Understanding chemical ordering in intermetallic clathrates from atomic scale simulations,* Chemistry of Materials **29**, 7554 (2017). doi:10.1021/acs.chemmater.7b02686.

[10] B. Sadigh and P. Erhart, *Calculations of excess free energies of precipitates via direct thermodynamic integration across phase boundaries,* Physical Review B **86**, 134204 (2012). doi:10.1103/PhysRevB.86.134204.

[11] M. Ångqvist, J. M. Rahm, L. Gharaee, and P. Erhart, *Structurally driven asymmetric miscibility in the phase diagram of W–Ti,* Physical Review Materials **3**, 073605 (2019). doi:10.1103/PhysRevMaterials.3.073605.

[12] A. H. Larsen, J. J. Mortensen, J. Blomqvist, I. E. Castelli, R. Christensen, M. Du\lak, J. Friis, M. N. Groves, B. Hammer, C. Hargus, E. D. Hermes, P. C. Jennings, P. B. Jensen, J. Kermode, J. R. Kitchin, E. L. Kolsbjerg, J. Kubal, K. Kaasbjerg, S. Lysgaard, J. B. Maronsson, T. Maxson, T. Olsen, L. Pastewka, A. Peterson, C. Rostgaard, J. Schiøtz, O. Schütt, M. Strange, K. S. Thygesen, T. Vegge, L. Vilhelmsen, M. Walter, Z. Zeng, and K. W. Jacobsen, *The atomic simulation environment — a Python library for working with atoms,* Journal of Physics: Condensed Matter **29**, 273002 (2017). doi:10.1088/1361-648X/aa680e.

[13] Data and code that was used to generate the results presented here is available via zenodo at doi:10.5281/zenodo.4287250.
